# The linkage of innate and adaptive immune response during granulomatous development

**DOI:** 10.3389/fimmu.2013.00010

**Published:** 2013-01-31

**Authors:** Toshihiro Ito, Judith M. Connett, Steven L. Kunkel, Akihiro Matsukawa

**Affiliations:** ^1^Department of Pathology and Experimental Medicine, Graduate School of Medicine, Dentistry and Pharmaceutical Sciences, Okayama UniversityOkayama, Japan; ^2^Department of Pathology, University of Michigan Medical SchoolAnn Arbor, MI, USA

**Keywords:** Notch signaling, Toll-like receptor, dendritic cell, T helper cell, innate immunity, acquired immunity

## Abstract

Granulomas represent a spectrum of inflammatory sequestration responses that may be initiated by a variety of agents, including non-infectious environmental factors and infectious microbial pathogens. Although this reaction is designed to be protective, the associated tissue injury is often responsible for a profound degree of pathology. While many of the mechanisms that sustain the development of the granuloma are enigmatic, it is accepted that the maintenance of this inflammatory process is dependent upon dynamic interactions between an inciting agent, inflammatory mediators, various immune and inflammatory cells, and structural cells of the involved tissue. The best studied of the host-dependent processes during granuloma development is the innate and adaptive immune response. The innate immune response by antigen-presenting cells [APCs; dendritic cells (DCs) and macrophages] is initiated quickly to protect from overwhelming pathogens, but with time, can also activate the adaptive immune response. APCs, essential regulators of the innate immune response, can respond to microbial ligands through Toll-like receptors (TLRs), which function in the recognition of microbial components and play an important role to link the innate and adaptive immune responses. CD4^+^ T helper (Th) cells are essential regulators of adaptive immune responses and inflammatory diseases. Recently, the Notch system has been shown to be an important bridge between APCs and T cell communication circuits. In the present review, we discuss recent findings that explore the mechanisms in the linkage of innate and adaptive immunity, including granulomatous formation though TLRs and Notch activation.

## Introduction

The granulomatous response is a complex host defense mechanism that has evolved to provide containment of infectious and/or environmental agents (Warren, 1976; El-Zammar and Katzenstein, 2007). The maintenance of this inflammatory process is dependent upon an inciting agent and the dynamic interactions amongst inflammatory mediators, various immune and inflammatory cells, and structural cells of the involved tissue (Chensue et al., 1994; Ulrichs and Kaufmann, 2006; Ramakrishnan, 2012). The best studied of the host-dependent processes during granuloma development are the innate and adaptive immune responses, which are characterized by specific immune cell populations each expressing a defining phenotype (Chiu et al., 2004; Raymond et al., 2007; Wolf et al., 2007; Carson et al., 2011).

The initial response to infection is the engagement of the host's innate immune system that triggers a rapid, multifaceted anti-infectious response involving the release of proinflammatory cytokines and eventually leads to the activation of the adaptive immune response (Kumar et al., 2009). The first line of defense is initiated when cellular pattern recognition receptors (PRRs) located on antigen-presenting cells (APCs) recognize pathogen-associated molecular patterns (PAMPs) (Guillot et al., 2005; Goodman et al., 2010). Recognition of PAMPs by PRRs rapidly triggers the innate immune response characterized by an array of antimicrobial immune responses through the induction of various inflammatory cytokines and chemokines (Kumar et al., 2009, 2011). Several families of PRRs, including Toll-like receptors (TLRs), Retonoic acid-inducible gene I (RIG-I)-like receptors, nucleotide-binding oligomerization-like receptors, and DNA receptors (cytosolic sensors for DNA) are known to play a crucial role in host defense (Kumar et al., 2009, 2011). Dendritic cells (DCs) and macrophages are professional APCs that can respond to pathogens through PRRs, which function in the recognition of infectious components and play an important role in both the innate and adaptive immune responses (Akira et al., 2006; Trinchieri and Sher, 2007). Over time APCs can activate the adaptive immune response to the invading pathogens by triggering T cell differentiation (Lukacs et al., 2008; Cuddapah et al., 2010). Recent data have indicated that the controlled expression of Notch receptor proteins on T cells is essential for normal T cell development and maturation (Osborne and Minter, 2007). The connection between PRRs and Notch pathways has helped to define the complex role of APCs in the regulation of T cell differentiation (Amsen et al., 2009b). We here review recent advances concerning the role of Notch signaling in T cell differentiation during infection and present our findings showing the role of the Notch system in linking innate and acquired immunity in inflammatory granulomatous response models.

## Type-1 and type-2 granuloma models in mice

**Figure 1:**
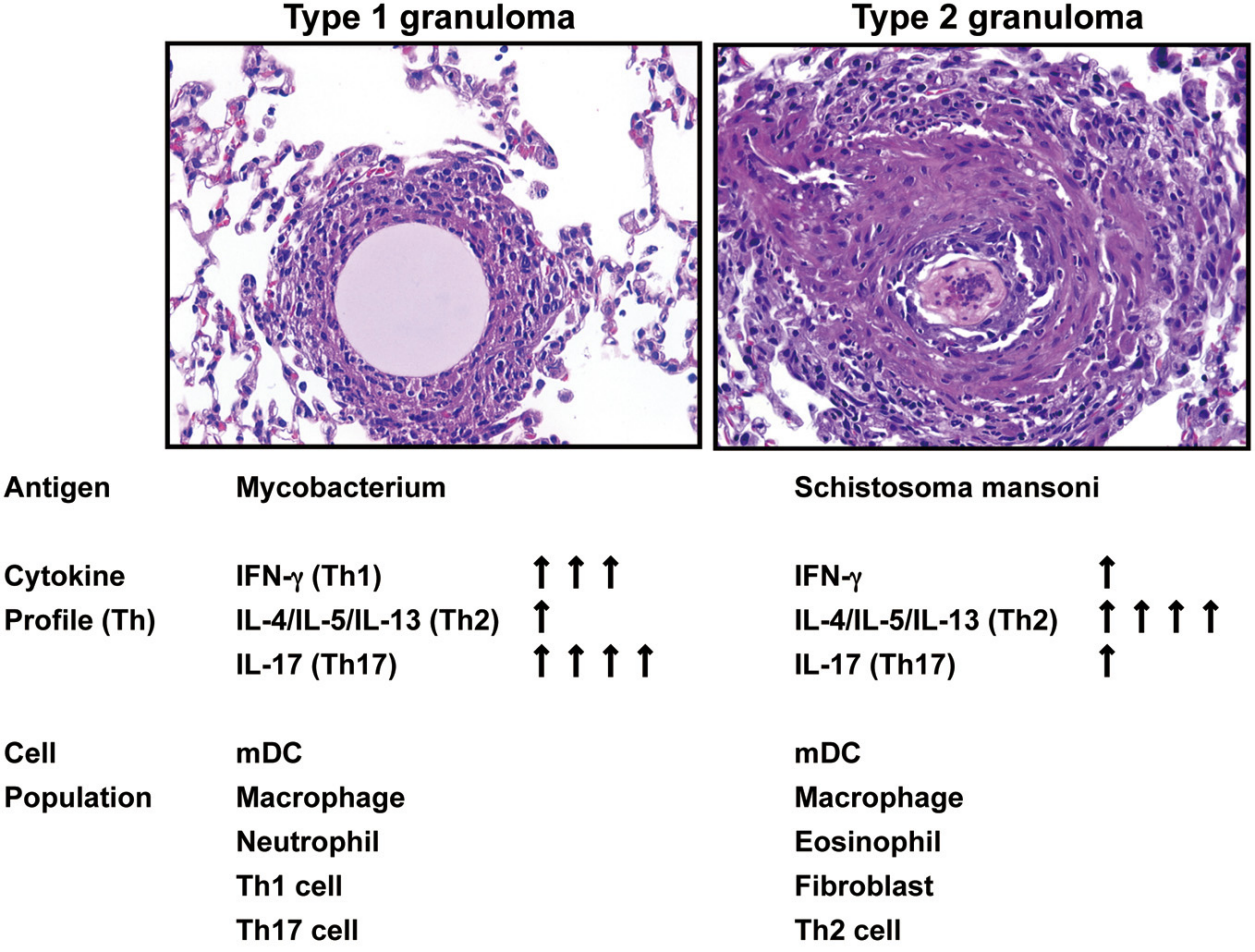
**Summary of Hematoxylin-and-eosin–stained sections showing the progression of type 1 and type 2 granuloma and the kinetic assessment of cytokine and cellular components**.

Data derived from a variety of models demonstrate that a number of inflammatory systems are involved in the induction of cytokines, which subsequently play a role in the initiation and maintenance of chronic experimental pulmonary inflammation (Raymond et al., 2007). However, the mechanistic contribution of various cytokines during the evolution, and more importantly, the maintenance of disease chronicity only recently have been addressed. For example, *in vivo* studies assessing the type-1 response elicited during the development of chronic lung granulomas induced by mycobacterial antigen have demonstrated that Interferon (IFN)-γ, Interleukin (IL)-12, and tumor necrosis factor (TNF) were necessary for lung lesion progression (Chensue et al., 1994). In contrast, type-2 experimental chronic lung granulomas initiated by complex antigens, including those derived from allergens (e.g., ovalbumin) and parasites (e.g., *Schistosoma mansoni*), were shown to be maintained by IL-4, IL-5, and IL-13, hallmarks of a type 2 response (Chensue et al., 1992; Ruth et al., 2000; Joshi et al., 2008). One of the most widespread and well-characterized type-1 granuloma diseases is tuberculosis. It has been estimated that one-third of the world's population is infected with *Mycobacterium tuberculosis*—resulting in more than 8.8 million cases of active tuberculosis with 1.4 million deaths globally in 2010 alone. We have established clinically relevant type 1 animal models with signature type-1 cytokine phenotypes. These models are established by the pre-sensitization of mice with either Mycobacterium species (BCG) or *Schistosoma mansoni* antigen followed by a pulmonary challenge with sized Sepharose beads coated with a known amount of tuberculin purified protein derivative (PPD) (Chensue et al., 1995; Ito et al., 2007, 2009a). We have also established a clinically relevant experimental type 2 model of inflammatory granuloma development by delivering *Schistosoma mansoni* eggs to the lungs, which release highly antigenic glycoproteins, referred to as Schistosoma egg antigen (SEA), that promote a dominant Th2 response (Chensue et al., 1992; Ruth et al., 2000; Ito et al., 2009b) (Figure 1). *Schistosoma mansoni* affects more than 80 million people worldwide, causing the disease intestinal schistosomiasis (Crompton, 1999; Hotez et al., 2008). It is the most widespread of the human-infecting schistosomes, and the host immune response to *S. mansoni* infection has been the most widely studied of the major schistosome species (Pearce and Macdonald, 2002; Schramm and Haas, 2010). These data offer the basis for continued studies to understand the mechanisms underlying these widespread diseases.

## Innate and acquired immunity in granuloma development

The typical granuloma contains mostly macrophages and DCs, surrounded by T lymphocytes, while myeloid DCs also have been found in the granulomas of tuberculosis patients and mouse tuberculin models (Ulrichs and Kaufmann, 2006; Ito et al., 2007; Silva Miranda et al., 2012). Of these cells, macrophages are the dominant cell type found in granulomas (Pieters, 2008), and these can be of two varieties: classically activated (M1) macrophages and alternatively activated (M2) macrophages, each of which have characteristic gene-expression profiles defined by markers linked to the stimulation conditions used to generate the subtype (Gordon and Taylor, 2005; Mantovani et al., 2007). Bacterial infection drives TLR engagement and IFN-γ expression skewing toward the M1 macrophage phenotype (pro-inflammatory and initiamicrobicidal), while *Schistosoma mansoni* infection drives IL-4 and IL-13 skewing toward the M2 macrophage phenotype (immunosuppressant and tissue repairers) (Herbert et al., 2004; Mosser and Edwards, 2008). In the granuloma, the presence of both types of macrophages may be required to maintain a balance between the pro- and anti-inflammatory response (Pieters, 2008). Although in comparison to macrophages, there are fewer numbers of DCs surrounding granulomas, the DCs play a critical role in antigen presentation, and produce large amounts of MHC-II and co-stimulatory molecules, initiating the linkage between innate and acquired immunity, which leads to T cell differentiation and activation (Wolf et al., 2007). T lymphocytes account for 15–50% of the leukocytes in mouse granulomas. About 60–70% of the T cells present are CD4^+^, 15–30% are CD8^+^ T cells, and there are also about 2%γδ T cells (Tsai et al., 2006; Silva Miranda et al., 2012). Of note, for example, the essential role of CD4^+^ T cells in the control of mycobacterial infection has been highlighted by many studies in knockout mice, and also in HIV patients (Ladel et al., 1995; Ito et al., 2007, 2009a; Geldmacher et al., 2012; Silva Miranda et al., 2012).

## TLRs for innate immunity

Innate immunity is the first line of host defense directed against invading pathogens and is designed to maintain host integrity (Si-Tahar et al., 2009). Innate immunity is activated immediately upon infection, is not antigen-specific, has no memory requirement, and requires large numbers of cells for pathogen recognition (Kabelitz and Medzhitov, 2007). Only if the invading pathogen is able to escape or overwhelm the innate response is acquired immunity activated. In some of these latter cases the host pro-inflammatory immune response becomes excessive, leading to pathological inflammation (Si-Tahar et al., 2009). Recent data suggest that the innate immune response initiated by the PAMP activation of specific TLRs can contribute mechanistically to maintaining the intensity and chronicity of the lung pathology associated with the developing granuloma (Ito et al., 2007, 2009b; Raymond et al., 2007). TLRs are named after their similarity to Toll, an essential receptor of the innate immune system against fungal infection, first discovered in Drosophila (Lemaitre et al., 1996). In recent years the family of mammalian TLRs expressed on APCs, namely DCs and macrophages, has been found to be key PRRs with central roles in the induction of the innate immune response. The principal functions of macrophages and DCs are phagocytosis and the elimination of microorganisms (Takeda and Akira, 2005). These cells also possess secondary functions including the production of cytokines, chemokines and chemotactic lipids, which direct certain circulating cells to migrate to the site of infection and participate in the elimination of pathogens. DCs also play an important role in communicating with and presenting antigens to lymphocytes, thus linking the innate and adaptive immune responses.

Microbial products, including mycobacterium antigen, activate specific TLRs, which in turn induce specific gene transcription resulting in the up-regulation and secretion of select chemokines and cytokines (Krutzik and Modlin, 2004; Ryffel et al., 2005; Jo et al., 2007). When activated, TLR specifically recruit adapter proteins [Myeloid differentiation factor 88 (MyD88), MyD88 adaptor-like (MAL), TIR domain-containing adaptor-inducing IFN-β (TRIF), and TRIF-related adaptor molecule (TRAM)], which are essential for the TLR gene transcription signaling cascade (Kawai and Akira, 2010; Kumar et al., 2011). Recent studies have provided new insights on how TLRs are involved in the recognition of specific pathogens, and have clarified their roles in both the innate and adaptive immune response (Goldstein, 2004). For example, mycobacterial components act as agonists for TLR, and mice that are deficient in the TLR adaptor molecule, MyD88, show an impaired response to mycobacterial antigens (Fremond et al., 2004). Although the response to mycobacterium antigens appears dependent on MyD88, the TLR involved has not been identified. All TLR, except TLR3, have at least one signaling pathway dependent on MyD88 (Kawai and Akira, 2010). The involvement of different TLRs, including TLR2, TLR4, TLR6, and TLR9 has been shown to recognize mycobacterium antigens in both mice and humans (Abel et al., 2002; Sugawara et al., 2003; Bafica et al., 2005; Motsinger-Reif et al., 2010). Specifically, TLR9 recognizes viral and bacterial CpG-DNA motifs, which when bound to TLR9 on macrophages and DCs, cause their activation (Jakob et al., 1998; Sparwasser et al., 1998; Hemmi et al., 2000). Further, TLR9 plays an important role in the regulation of the mycobacteria-induced T helper (Th) responses during *M. tuberculosis* infection *in vivo* (Bafica et al., 2005; Ito et al., 2007). The activation of TLR9 requires the uptake of microbes (or synthetic CpG oligodeoxynucleotides) within endosomes, the formation of DNA:TLR9 complexes within the endocytic vesicles, and the subsequent acidification and maturation of the endosomes (Latz et al., 2004; Wagner, 2004; Yasuda et al., 2005). Some observations support a role for TLR9 in the host response to lung infectious pneumonia induced by a variety of microbes including *Mycobacterium tuberculosis* (Bafica et al., 2005; Bhan et al., 2007; Kleinnijenhuis et al., 2011). We will return to this point later when we review our own recently published data.

## Notch system for acquired immunity

Pathogens such as bacteria, helminths, fungi, and viruses are recognized by and activate APCs, which in turn activate CD4^+^ Th cells (Kumar et al., 2011). The Th cells drive adaptive immunity and induce specific responses against the infecting microbes (Trinchieri and Sher, 2007). It has been shown that the different types of APCs and their availability to display particular cytokine production profiles, pathogen recognition receptors (PRRs), and co-stimulatory molecules are key determinants for Th differentiation (Carballido et al., 2006).

In addition, it has been shown that Notch proteins are also important in the induction of Th responses (Amsen et al., 2009a; Radtke et al., 2010). Notch is a heterodimeric cell-surface receptor family (Notch 1–4) that is involved in a broad range of differentiation processes (Bray, 2006). Notch family members are composed of an extracellular ligand-binding domain that is non-covalently associated with a single-pass transmembrane domain. The Notch signaling pathway regulates many aspects of embryonic development, as well as differentiation processes and tissue homeostasis in multiple adult organ systems. There are two distinct families of Notch ligands in mammals, known as the Delta-like ligands (consisting of Dll1, Dll3, and Dll4) and the Jagged ligands (Jagged1 and Jagged2); both Dll and Jagged proteins trigger the canonical Notch signaling pathway wherein, binding of a ligand to a Notch receptor results in the cleavage of the receptor at a site in the transmembrane portion (Ehebauer et al., 2006). Upon binding by either Dll or Jagged ligands, Notch undergoes proteolytic cleavage catalyzed by Adam proteases and the γ-secretase complex, leading to the translocation of the notch intracellular domain (N-ICD) into the nucleus. N-ICD interacts with the transcriptional repressor, recombination-signal-binding protein for immunoglobulin-kJ region (RBP-J). The N-ICD interaction with RBP-J and also recruits Mastermind (MAML) protein. The new transcriptional complex of N-ICD-RBP-J-MAML converts RBP-J from a repressor to a transcriptional activator (Dallman et al., 2003; Ehebauer et al., 2006). Regulation of Notch signaling is associated with several human disorders, including cancer. It has been well documented in cancer studies that the Notch pathway influences stem cell maintenance, development and cell fate, and that it also promotes cell survival, angiogenesis, and treatment resistance in numerous cancers, making it a promising target for cancer therapy (D'Souza et al., 2008). More recently, it has become evident that Notch signaling plays an important role within the hematopoietic and immune systems. In the mature immune system, the Notch pathway has been shown to be involved in regulating Th1, Th2, and Th17 cell lineage choices for T cells, each of which is characterized by the production of distinct cytokines and effector functions (Radtke et al., 2010; Ito et al., 2012). For example, Th1 cells produce IFN-γ and target intracellular pathogens, while Th2 cells secrete IL-4, IL-5, and IL-13 and target helminthes (Kapsenberg, 2003; Ito et al., 2009b). In the presence of functional MyD88, an adaptor molecule of TLR, PAMP (derived from bacteria, viruses, or other TLR-ligands) binding to TLR upregulates Dll1 or Dll4 on APCs, which causes the differentiation of naïve Th cells to a Th1 phenotype (Maekawa et al., 2003; Amsen et al., 2004, 2009b). On the other hand, the differentiation of naïve Th cells to a Th2 phenotype occurred in the absence of functional MyD88 when Jagged was constitutively expressed on APCs (Amsen et al., 2009b). Thus data suggest that Dll1 and Dll4 cause Th1 skewing while Jagged causes Th2 skewing. Moreover, recent findings described IL-17-producing Th17 cells that play an essential role in host defense via protection against extracellular bacterial and fungus, while also recruiting neutrophils to the site of infection (Korn et al., 2009; Pappu et al., 2011). Neutrophils play a key role in the front-line defence against invading pathogens by phagocytosis as well as by recruiting other inflammatory cells (Lowe et al., 2012). Moreover, Th17 cells not only defend against bacterial and fungus infections but also are involved in autoimmune disease, allergic responses, and cancer (Wilke et al., 2011). However, how Dll- and Jagged-expressing APCs differ in their ability to induce Notch signaling and discriminate amongst their various described functions is unclear. More in-depth studies using mice that are deficient in each Notch and Notch ligand protein will provide data about how specific Notch ligands (Delta and Jagged ligands) control the different types of T cell responses during physiological conditions. Validation of the *in vitro* data will require a number of *in vivo* studies using diverse granulomatous models, including bacterial, parasitic, and fungal, as well as autoimmune models.

## Mycobacterial granulomatous response though TLRs and notch activation

**Figure 2:**
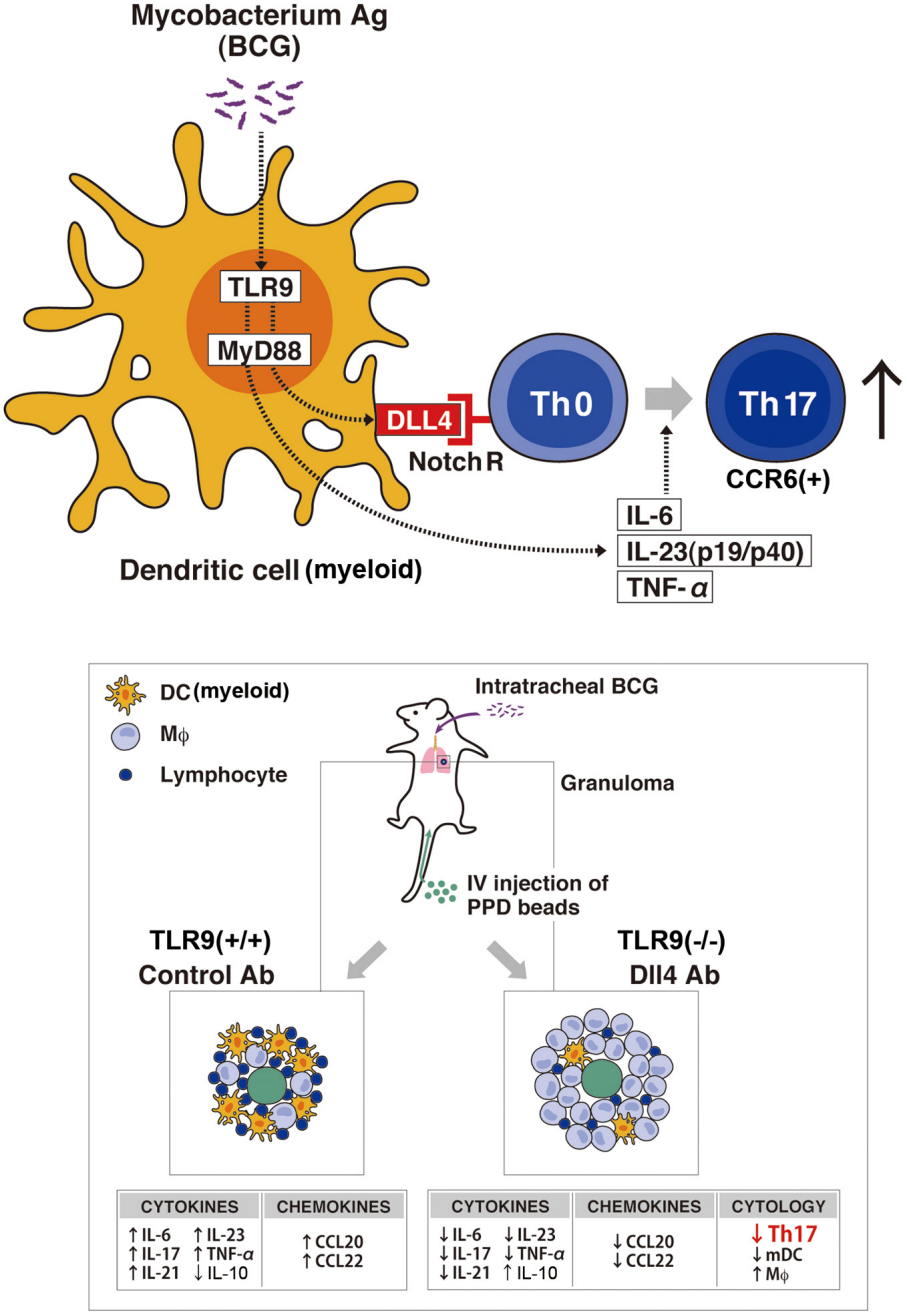
**Schematic representation of the TLR9-Notch ligand (dll4) on *Mycobacterium*-dependent granuloma formation.** Myeloid DCs (mDCs) play an important role in inducing the differentiation of Th17 cells through the TLR9 effector pathway that upregulates the Notch ligand dll4. *In vivo* granuloma formation induced by BCG/*Mycobacterium* Ag demonstrates larger granuloma formation in TLR9-knockout mice (TLR9^−/−^) with decreased numbers of Th17 cells (CCR6+) and mDCs in the lungs when compared with lung granulomas from WT mice. Further, TLR9^−/−^ mice showed an increase in IL-10 with a concomitant decrease in Th17 cell-related cytokines (IL-17, IL-6, IL-21, IL-23, and TNF-α) and a decrease in the levels of the chemokines CCL20 and CCL22, important for DC migration, compared with levels in WT mice. The decreased expression of dll4 and the perturbation of the indicated cytokine and chemokine expression levels led to the abrogation of the Th17 phenotype in the Anti-Dll4 Ab treated mice with the concomitant increase in granuloma size. Accompanying these phenomena, there was a decrease in Th17 cells and mDCs in the lungs of Anti-Dll4 Ab treated mice and an increase in lung macrophages.

While the mechanism of granuloma formation is unclear, this distinct cellular response is considered a histologic hallmark for a protective immune response, involving both innate and adaptive immunity. TLR9 is known to play a role in the regulation of Th1 responses (Bafica et al., 2005; Huang et al., 2005); thus, we have investigated the role of TLR9 in granuloma formation during challenge with mycobacterium antigens and demonstrated that mice deficient in TLR9 had increased granuloma formation with a dramatically altered cytokine phenotype. While Th1 cytokine levels of IFN-γ and IL-12 in the lungs were decreased in TLR9^−/−^ mice when compared to wild-type mice, Th2 cytokine levels of IL-4, IL-5, and IL-13 were increased in these knockout mice (Ito et al., 2007). This response suggests that IL-12 production is TLR9-MyD88 pathway dependent. More recently, our group showed the first analysis of cell-mediated Th17-related pulmonary mycobacterial Ag-elicited granuloma formation in TLR9^−/−^ mice and defined a role for TLR9 in the induction of both Notch ligand Dll4 and Th17 expression using both *in vivo* and *in vitro* approaches (Ito et al., 2009a). Our studies demonstrated that TLR9^−/−^ mice exhibited significantly larger granuloma formation, following an impaired Th17-like response with decreased expression of Dll4 on DCs from TLR9^−/−^ mice compared with WT mice. Dll4 was the primary Notch ligand upregulated by mycobacterial infection of DCs in WT mice. When Dll4 was specifically blocked *in vivo* using anti-Dll4 Ab during mycobacteria-induced pulmonary granuloma formation, Th17 cellular responses were significantly inhibited and larger granulomas were observed. Moreover, in *in vitro* experiments, anti-dll4 antibody specifically blocked IL-17 production by CD4^+^ T cells, while overexpression of Dll4 augmented IL-17 production by CD4^+^ T cells, suggesting that Dll4 plays an important role in promoting Th17 activity during a mycobacterial challenge. In contrast, the observed histologic alterations in lung granuloma development in TLR9^−/−^ mice also coincided with a significant decrease in lung myeloid DCs, which are crucial to the differentiation of Th17 cells, as well as decreased levels of dll4 on DCs when compared with wild-type mice. This impaired migration of lung myeloid DCs in granuloma was attributed to decreased production of chemokine CCL20. Chemokines constitute a family of structurally related chemotactic cytokines that direct the migration of leukocytes throughout the body under both physiological and inflammatory conditions (Matsushima, 2000). CCL20 and its receptor CCR6 play a role in the recruitment of immature DCs and their precursors to sites of potential antigen entry (Schutyser et al., 2003). Thus, the lower expression of CCL20 during mycobacterial challenge in either TLR9-deficient mice or anti-dll4–treated lungs might contribute to the observed decreased DC numbers during pulmonary granuloma formation. In addition, Th17 cells induced *in vivo* in normal mice via homeostatic proliferation express CCR6 as well as CCL20 (Hirota et al., 2007). Our data show that lower CCL20 expression in lungs from TLR9^−/−^ mice and in lungs with anti-dll4 Ab is correlated with not only impaired DC migration but also reduced numbers of Th17 cells in lungs during mycobacteria induced pulmonary granuloma formation (Ito et al., 2009a) (Figure 2).

## Concluding remarks

Our data suggest that an understanding of Dll4 regulation of Th17 responses through Notch may provide mechanistic approaches for modifying and controlling the immune response induced by the Th17 phenotype. Moreover, a number of studies have demonstrated that Notch proteins are important in the induction of Th1 responses. In the presence of functional MyD88, PAMP binding to TLR up-regulates Dll4, which causes the differentiation of naïve Th cells to a Th1 phenotype. In addition, when Dll ligands are overexpressed on APCs or are cross-linked as fusion proteins, they also promote Th1 cell differentiation (Maekawa et al., 2003). Our recent study also revealed that Dll1 expression on macrophages is dependent on type-I IFN pathways, and is critical for protection against influenza virus A (H1N1) infection (Ito et al., 2011). Further, another of our recent findings indicated that Notch ligand Dll4 caused an increase in the expansion of Th2 memory cells and a decrease in effector cell proliferation in our experimental type 2 model of inflammatory granuloma development via the embolization of *Schistosoma mansoni* eggs to the lungs (Schaller et al., 2010). This study suggests that the Notch pathway also contributes to the different responses of memory and T effector (Teff) cells to Notch ligands, it also has been well established that Notch ligands can have different effects on T cell differentiation, depending on the immune environment including the local cytokine environment. However, the Notch system may be an even more complicated system, as these signaling pathways also contribute to multiple lineage decisions of developing lymphoid and myeloid cells. Moreover, the cellular constituents of the lung immune system are diverse and include not only leukocytes, such as macrophages, DCs, neutrophils, mast cells, and lymphocytes, but also, it has been shown that epithelial cells and fibroblasts play critical roles in the lung defense system. Further knowledge of the regulation of the Notch system in these cells and understanding the contextual interactions between these populations may provide mechanistic approaches for modifying and controlling the immune response during granulomatous diseases in clinically relevant translational studies. A better understanding of the regulation of the Notch system might contribute novel therapeutic approaches for granulomatous diseases.

### Conflict of interest statement

The authors declare that the research was conducted in the absence of any commercial or financial relationships that could be construed as a potential conflict of interest.
